# Development and application of a direct method to observe the implant/bone interface using simulated bone

**DOI:** 10.1186/s40064-016-2116-6

**Published:** 2016-04-21

**Authors:** Yoko Yamaguchi, Makoto Shiota, Masaki FuJii, Michi Sekiya, Masahiko Ozeki

**Affiliations:** Department of Implant Dentistry, School of Dentistry, Showa University, 2-1-1 Kitasenzoku Ota-ku, Tokyo, 145-8515 Japan; Division of Oral Health Sciences, Department of Masticatory Function Rehabilitation, Oral Implantology and Regenerative Dental Medicine, Graduate School, Tokyo Medical and Dental University, 1-5-45 Yushima, Bunkyo-ku, Tokyo, 113-8510 Japan; Dental Implant Clinic, Dental Hospital, Tokyo Medical and Dental University, 1-5-45 Yushima, Bunkyo-ku, Tokyo, 113-8510 Japan; Dental Implant Center, Showa Dental Hospital, 2-1-1 Kitasenzoku Ota-ku, Tokyo, 145-8515 Japan

**Keywords:** Primary stability, Implant design, Artificial bone, Implant/bone interface

## Abstract

**Background:**

Primary stability after implant placement is essential for osseointegration. It is important to understand the bone/implant interface for analyzing the influence of implant design on primary stability. In this study rigid polyurethane foam is used as artificial bone to evaluate the bone–implant interface and to identify where the torque is being generated during placement.

**Methods:**

Five implant systems—Straumann-Standard (ST), Straumann-Bone Level (BL), Straumann-Tapered Effect (TE), Nobel Biocare-Brånemark MKIII (MK3), and Nobel Biocare-Brånemark MKIV (MK4)—were used for this experiment. Artificial bone blocks were prepared and the implant was installed. After placement, a metal jig and one side artificial bone block were removed and then the implant embedded in the artificial bone was exposed for observing the bone–implant interface. A digital micro-analyzer was used for observing the contact interface.

**Results:**

The insertion torque values were 39.35, 23.78, 12.53, 26.35, and 17.79 N cm for MK4, BL, ST, TE, and MK3, respectively. In ST, MK3, TE, MK4, and BL the white layer areas were 61 × 103 μm^2^, 37 × 103 μm^2^, 103 × 103 μm^2^ in the tapered portion and 84 × 03 μm^2^ in the parallel portion, 134 × 103 μm^2^, and 98 × 103 μm^2^ in the tapered portion and 87 × 103 μm^2^ in the parallel portion, respectively.

**Conclusions:**

The direct observation method of the implant/artificial bone interface is a simple and useful method that enables the identification of the area where implant retention occurs. A white layer at the site of stress concentration during implant placement was identified and the magnitude of the stress was quantitatively estimated. The site where the highest torque occurred was the area from the thread crest to the thread root and the under and lateral aspect of the platform. The artificial bone debris created by the self-tapping blade accumulated in both the cutting chamber and in the space between the threads and artificial bone.

## Background

Primary stability after implant placement is essential for osseointegration. Aside from the patient’s bone quantity and density, primary stability may also be affected by the implant design, including surface topography, and by the technique used to prepare the insertion socket (Friberg et al. 1995; Chiapasco et al. 1997; Javed and Romanos 2010). To analyze the influence of implant design on primary stability, it is also important to understand the bone/implant interface. Conventionally, microscopy of animal tissue samples (Orsini et al. 2010; Ericsson et al. 1994; Duyck et al. 2001; Shalabi et al. 2006; Marin et al. 2008) or radiographic analyses have been used (Akkocaoglu et al. 2005). The former focused on the morphology of the interface only after osseointegration had occurred, whereas the latter displayed low resolution, making it difficult to standardize (Meredith 1998) and provide an accurate evaluation. In an attempt to solve these issues, a novel method was developed to observe the implant/simulated bone interface using an artificial bone.

There are a number of reports investigating the properties of rigid polyurethane foam (PUF) for use as artificial bone (Thompson et al. 2003; Palissery et al. 2004; Szivek et al. 1993; Patel et al. 2008; Calvert et al. 2010). PUF is currently used as a standard orthopedic mechanical test material by the American Society for Testing and Materials (ASTM) (ASTM 2008). For mechanical testing of bone screws, the Japanese Industrial Standards recommend PUF to simulated bone, as it reproduces the dynamic characteristics of both cortical and cancellous bone (Doe 2009). The Solid Rigid Polyurethane and Cellular Rigid Polyurethane comply with the ASTM standard F1839, indicating that the materials are suitable for simulating bone to test orthopedic devices and instruments. In recent years, PUF has been used to simulated bone in dentistry for the retention of dental implants (Tabassum et al. 2009; Chong et al. 2009; Kim and Lim 2011; Kim et al. 2011; Ahn et al. 2012; Yamaguchi et al. 2015), as well as to investigate the retention of mini-implants (Lim et al. 2008; Kim et al. 2008). In this study, PUF was used evaluate the implant/simulated bone interface and to identify where the torque values is being generated during implant placement.

## Methods

### Implants

The types of implants used for this experiment and their design characteristics are summarized in Table 1. Standard RN (ST) and Brånemark MKIII (MK3) implants are parallel design. The outer profile of the Bone Level RC (BL) implant is entirely parallel, although the inner profile is tapered only in the neck region and the thread height is reduced. For the Tapered Effect RN (TE) implant, the apical portion is parallel and the inner and outer profiles display the same taper in the neck region. The Brånemark MKIV (MK4) implant has a gentle taper along the entire inner and outer profile, with a cutting chamber. ST, BL, and TE are considered non-self-tapping implants, whereas MK3 and MK4 are self-tapping implants with double-threaded.

**Table 1:** Implants used in this study

Implant system	Length (mm)	Diameter (mm)	Pitch (mm)	Lead (mm)	Code	Manufacturer
Standard RN	10	4.1	1.2	1.2	ST	Straumann
Bone level RC	10	4.1	0.8	0.8	TE	Straumann
Tapered effect RN	10	4.1	0.8	0.8	TE	Straumann
Brånemark MKIII	10	3.75	0.6	1.2	MK3	Nobel Biocare
Brånemark MKIV	10	4.0	0.6	1.2	MK4	Nobel Biocare

### Direct observation method

The mean bone mineral density for the posterior maxilla is 0.31 g/cm^3^ ( 2015). The densities of used PUF (0.32 g/cm^3^) were density of similar in type 4 bone density, and the following physical properties: 8.4 MPa compressive strength, 5.6 MPa tensile strength, 4.3 MPa shear strength, and a coefficient of elasticity of 284 GPa (Solid Rigid polyurethane Form 20 pcf; Sawbone; pacific Research Laboratories Inc., USA) (Doe 2015).

**Fig. 1:**
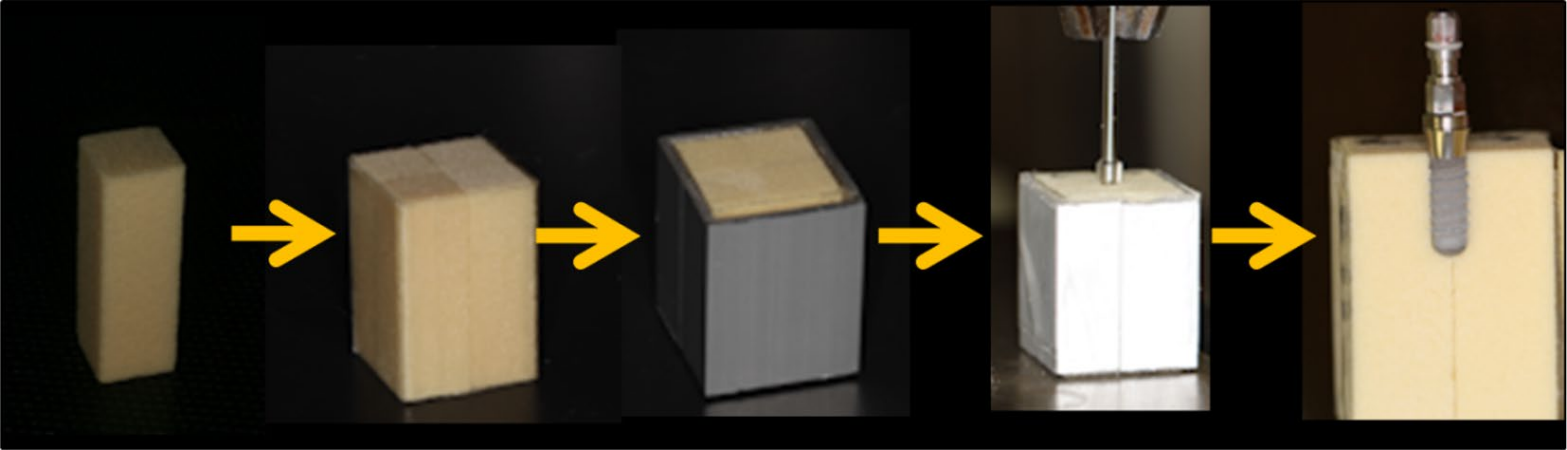
Flow of a divided block specimen. Four divided block specimen was assembled by tape. Their circumference was fixed with metal jigs and the implant was inserted according to the manufacturer instructions. After the placement, the metal jigs and two of the divided block were removed carefully

A divided block specimen is assembled by following procedures. Four pieces of prismatic simulated bone of 1 cm × 1 cm × 3 cm were prepared, they were fixed with tape beforehand, and their circumference was fixed with metal jigs. The insertion socket was prepared in the center of the column using manufacture protocol (ASD-360, Ashina, Hiroshima, Japan), avoiding movement. Each implant was installed with a load of 500 g and a speed of 15 rpm (Fig. 1). The maximum insertion torque-time curve during placemen was recorded using a torque analyzer (TRQ-5DRU, PC Torque Analyzer, Vectrix, Tokyo, Japan).

After implant placement, the metal jigs and two of the simulated bone were removed carefully, the implant body was exposed. A digital micro analyzer (VHX-1000, Keyence, Osaka, Japan) and image analysis software (PopImaging, Digital Being Kids, Kanagawa, Japan) were used for observation of the implant/simulated bone interface and for image analysis, respectively. The observation of the white layer was carried out only at representative specimen that closes to average value of IT. Regression analysis examining the correlation between the area of the white layer and Torque raise rate (JMP, SAS Institute Japan, Tokyo, Japan).

## Results

**Fig. 2:**
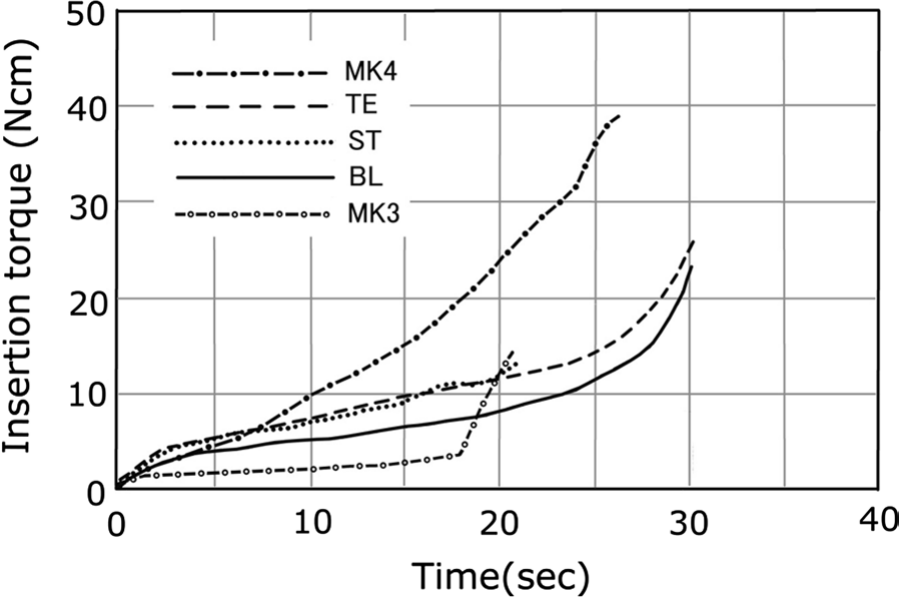
Torque-time curves for average insertion value of MK4, TE, ST, BL, MK3 implant

The insertion torque-time curves for each implant are illustrated in Fig. 2.

**Fig. 3:**
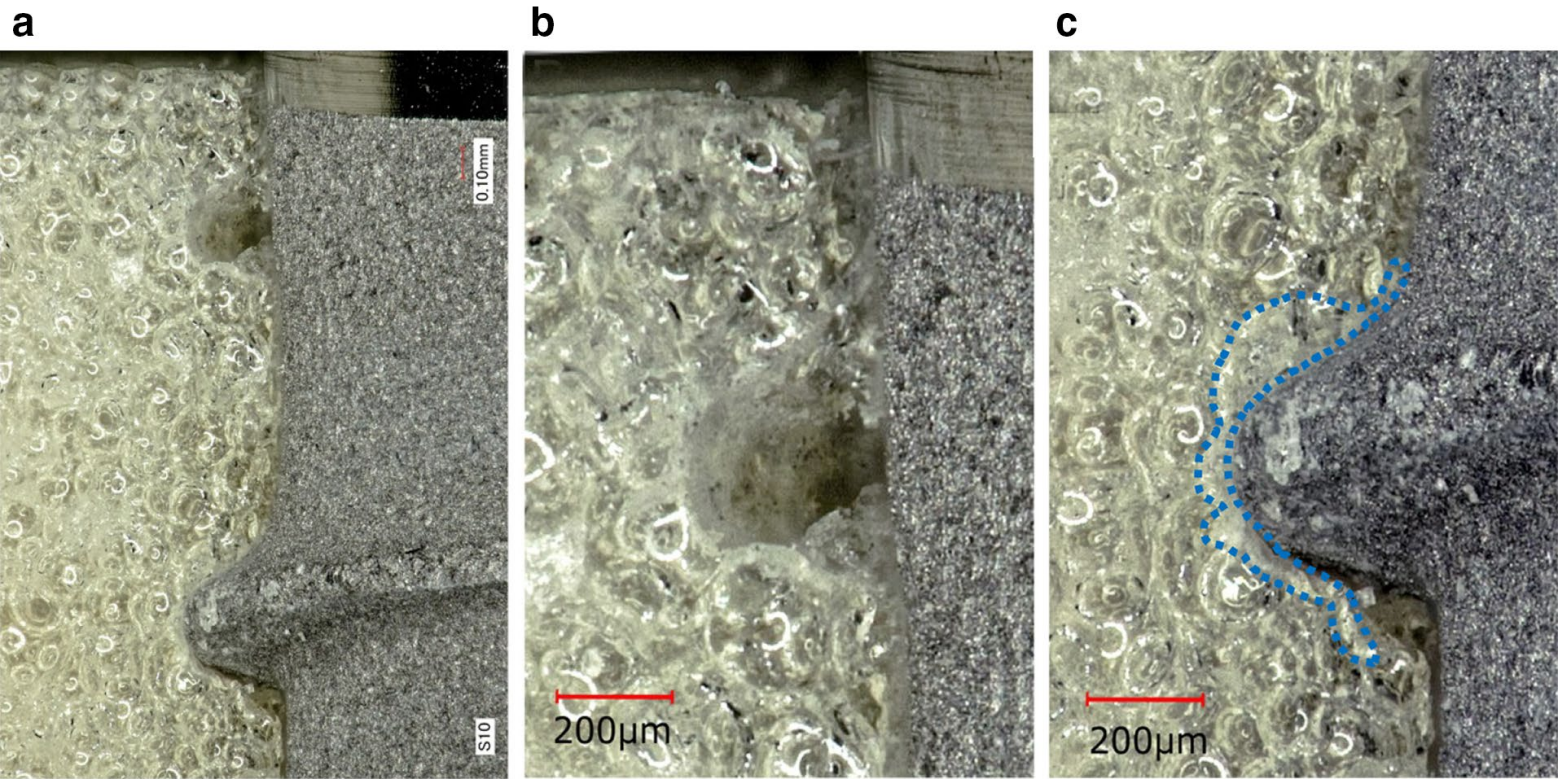
Representative photographs of ST/simulated bone interface. Photographs of **a** and **b** were taken from the neck of the implant, showing the tapped channel (**a**: magnification, ×30, **b**: magnification, ×40). A photograph of **c** was taken from the middle of the implants, showing the *white layer* (*dotted line*) (magnification, ×40)

Implant/simulated bone interface. In ST (Fig. 3a, b), a compressed channel was observed in the implant neck. The compressed channel had smaller morphology when compared with the threads, and a subtle circular-shaped white layer could be observed around the channel. Figure 3c shows a white layer around the thread crests. The upper surface of the threads engaged the artificial bone, although a gap could be seen between the under thread and the thread root. The area of the white layer was approximately 61 × 103 μm^2^.

**Fig. 4:**
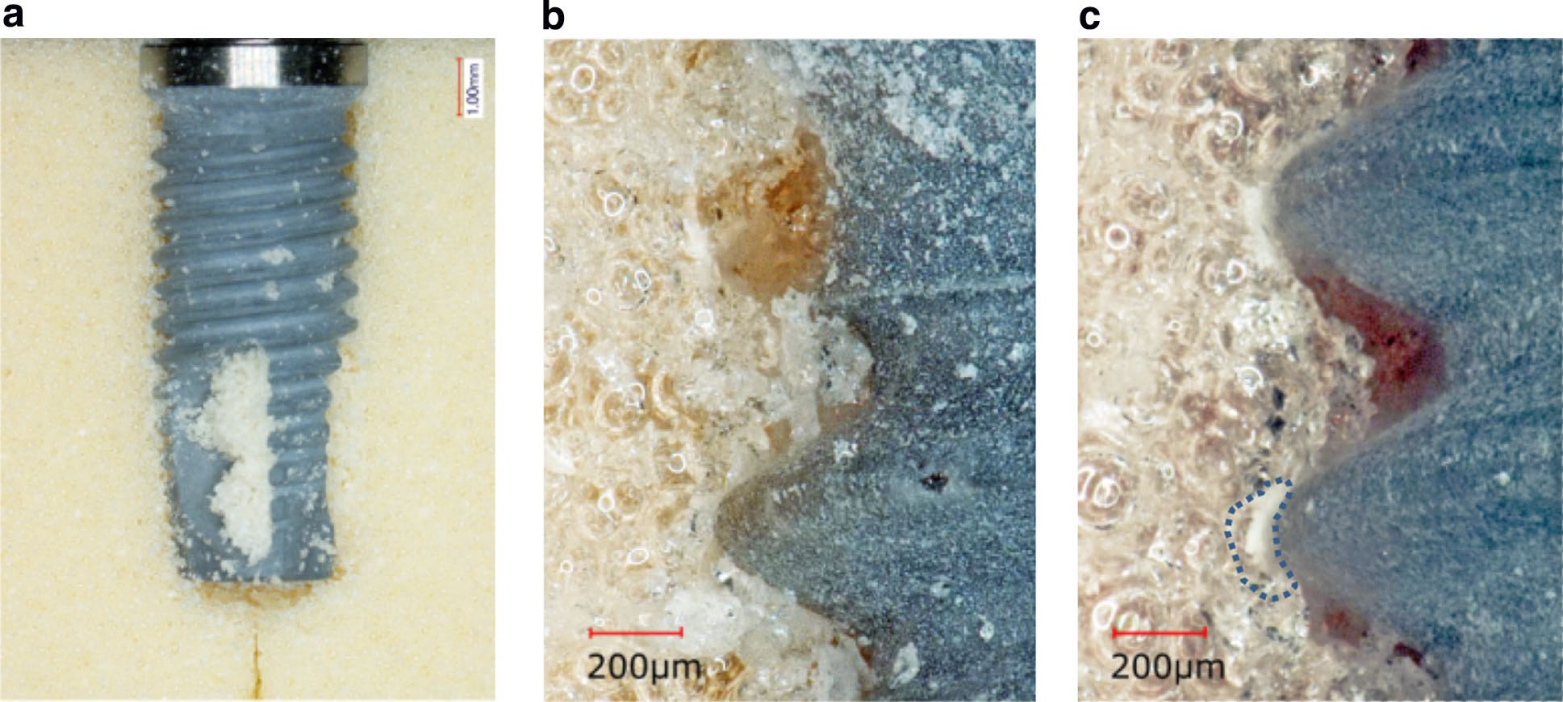
Representative photographs of MK3/simulated bone interface. Debris was scattered overall implant body and the cutting chamber (**a**: magnification, ×3). A photograph of **b** was taken from the neck of the implant, showing the tapped channel (magnification, ×30). A photograph of **c** was taken from the middle of the implant, showing the space between the under implant threads to the root (magnification, ×30)

In MK3, a gap between the tapped channel and the threads was also observed (Fig. 4a, b). The debris regarded as artificial bone chips were scattered along the implant surface and observed abundantly in the cutting chamber. A white layer could be observed around the threads, and debris could easily be removed by air-blowing. The thread crests closely engaged the artificial bone, although a gap could be seen under to the threads extending to the thread root. The white layer on the thread crest was 37 × 103 μm^2^ (Fig. 4c).

**Fig. 5:**
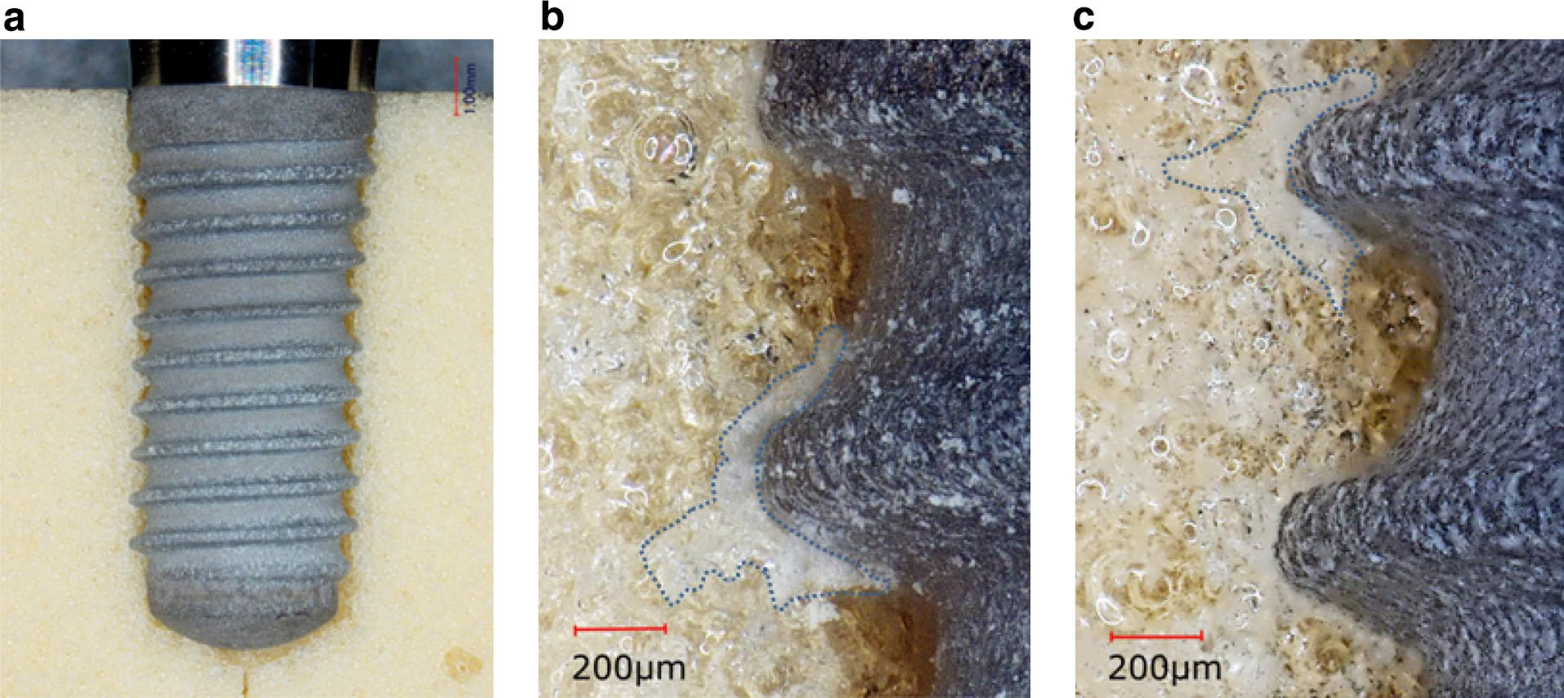
Representative photographs of TE/simulated bone interface. A photograph of **a** was showing the space between threads (magnification, ×5). A photograph of **b** was taken from the neck of the implants (tapered portion) (magnification, ×40) and **c** was taken from the middle of the implant, both showing the *white layer* (*dotted line*) and the space between the under implant threads to the root (magnification, ×40)

In TE, a gap was observed between the inner profile of the implant and the artificial bone (Fig. 5a). In the tapered portion, approximately half of the thread height engaged the artificial bone and a circular-shaped white layer were observed around the thread ridge (Fig. 5b, c). The upper threads closely engaged the artificial bone, although a gap could be seen between the under surface of the threads and the threads root. The white layer was 103 × 103 μm^2^ in the tapered portion (B) and 84 × 103 μm^2^ in the parallel portion (C).

**Fig. 6:**
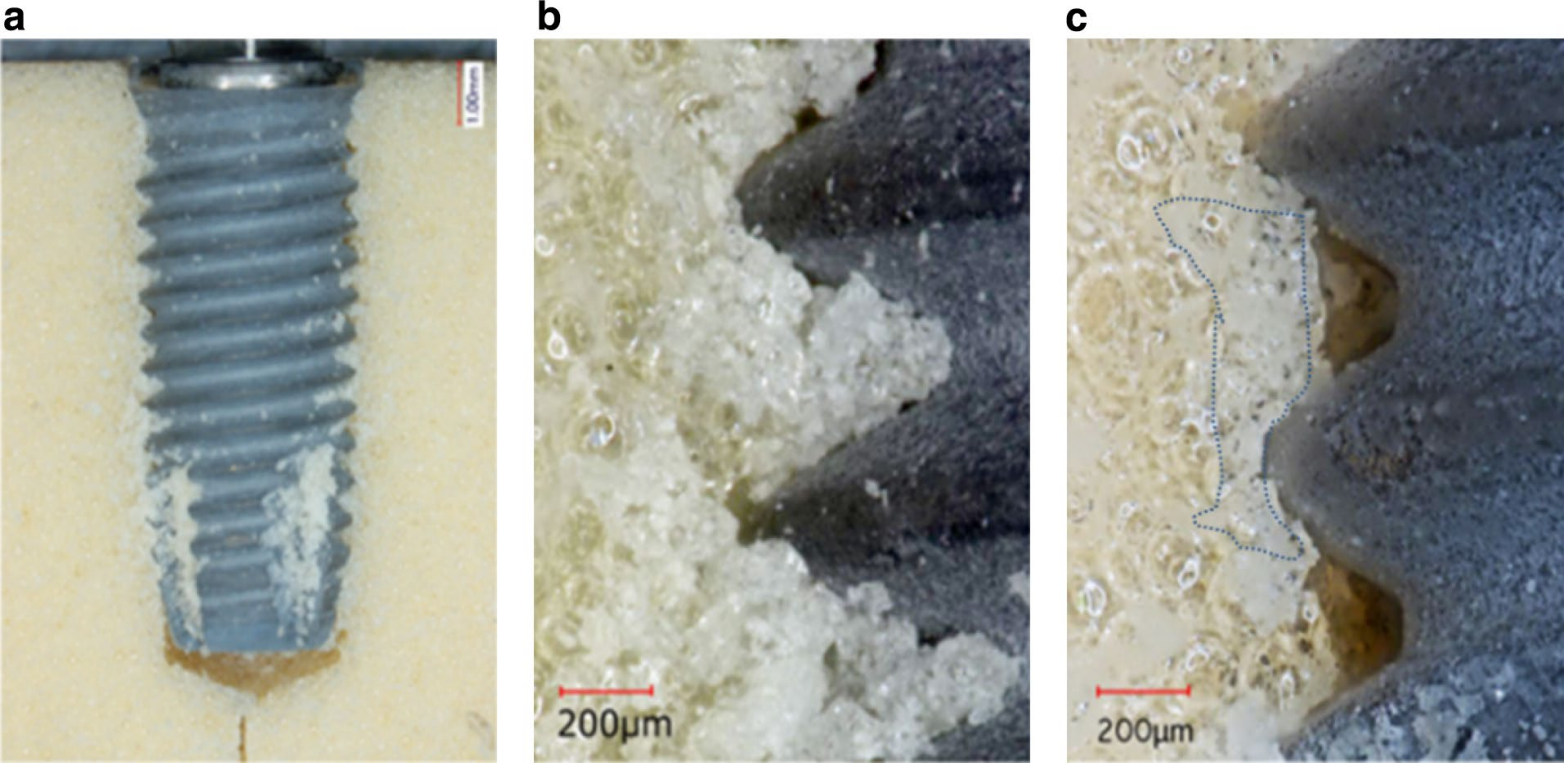
Representative photographs of MK4/simulated bone interface. Debris was scattered overall implant body and the cutting chamber (**a**: magnification, ×4). A photograph of **b** and **c** was taken from the middle of the implant (magnification, ×40), and **b** was showing debris in the threads root, and after air-blowing of **c** was showing the *white layer* (*dotted line*), the space realized thread root (magnification, ×40)

In MK4, slight gaps were observed along the entire implant and artificial bone interface (Fig. 6a). The cutting debris on the implant surface and thread root (Fig. 6b). The debris was removed and a gap was then observed around the thread root, and white layers were observed around both the thread crest and root. The white layer was 134 × 103 μm^2^ (Fig. 6c).

**Fig. 7:**
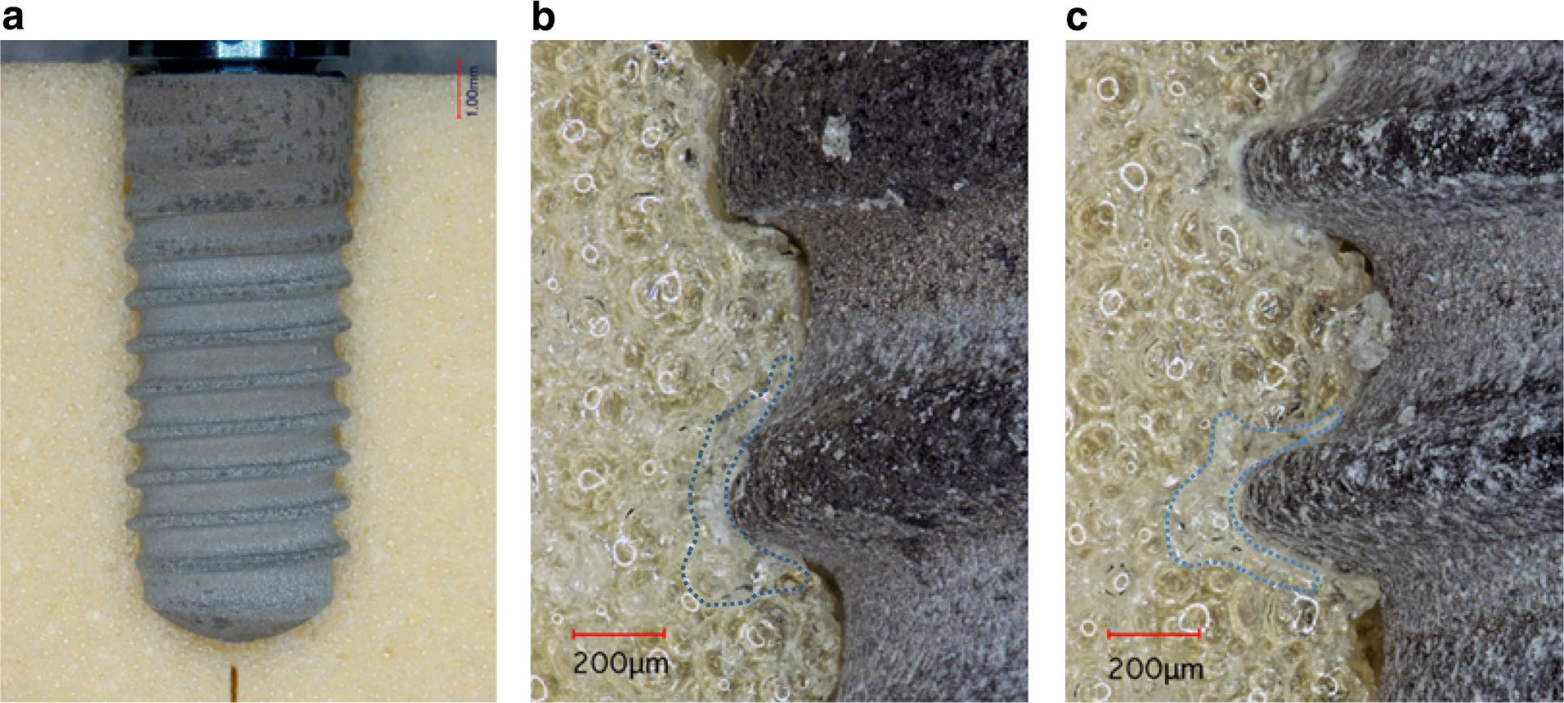
Representative photographs of BL/simulated bone interface. A photograph of **a** was showing the tapered portion engaged the simulated bone (magnification, ×5). A photograph of **b** was taken from the neck of the implant, showing the *white layer* (*dotted line*) (magnification, ×40). A photograph of **c** was taken from the middle of the implants, showing the *white layer* (*dotted line*) (magnification, ×40)

In BL, the upper thread surface closely engaged the artificial bone, and a gap was observed the thread root (Fig. 7a, b). The white layer surrounding the threads was 98 × 103 μm^2^ in the tapered portion (B) and 87 × 103 μm^2^ in the parallel portion (C).

Table 2 shows the white layer corresponds with the torque value. Regression analysis examining the correlation between the white layer area and torque raise rate, the correlation coefficient is 0.617566, slope 0.019372, intercept 0.60584 is obtained, a positive correlation was observed.

**Table 2:** Area of white layer and torque rise rate of each implant

Implant	Portion	Area of white layer (×103 μm^2^)	Torque rise rate (N cm/s)
ST	Parallel	61	0.45
MK3	Parallel	37	0.13
TE	Parallel	84	0.45
BL	Parallel	87	0.35
TE	Taper	103	2.31
MK4	Taper	134	1.32
BL	Taper	98	2.45

## Discussion

The bone density of used PUF was similar to other reports (Tabassum et al. 2009; Bayarchimeg et al. 2013), and the PUF is homogeneous consistency and eliminate anatomical or structural differences of biological samples to IT evaluate better the effects of the implant design (Tabassum et al. 2009). Furthermore, PUF exhibit stress–strain behaviors similar to trabecular bone (Calvert et al. 2010). The novel direct observation method devised for this study enables the center of the implant to be exposed without becoming destruction of the specimen; therefore, the implant/artificial bone interface could be observed successfully.

The insertion torque values provides more accurate data for the retention of an implant (Friberg et al. 1995; Homolka et al. 2002; Sakoh et al. 2006; Atsumi et al. 2007). The implant design affects the insertion torque values (O’Sullivan and Sennerby 2000). This study revealed that the white layer corresponds with the insertion torque to some degree. Differences in insertion torque value were related to differences in the morphology of the white layer. The relationship between the white layer area of the parallel and tapered sections with torque raise rate. The white layer area remains small when the implant design is parallel and torque increases gradually. The white layer area remains wide when a tapered design is used and the torque increases suddenly. A limitation of the direct method used to observe the implant/artificial bone interface is disturbance and spreading of the blocks when the insertion torques becomes large. Therefore, it is necessary to securely fix the blocks assembly using metal jigs and to ensure that the specimen is correctly placed by measuring the insertion torque values.

The white layers were observed around the implant threads in all specimens. The area of the white layers varied, and included the thread crests, circular-shaped sites around the thread ridges, and thread roots. Some areas were belt-shaped with a constant width and in other areas, the white layer spread deeply from the crest of the threads.

The comparing the IT of self-tapping and non-self-tapping implants were resulted the self-tapping implant higher insertion values than with non-self-tapping implants in a clinical study (Marković et al. 2013). The conical Implant compared with cylindric screw-type implant resulted in higher primary stability in soft bone (Sakoh et al. 2006). The primary stability of five implant designs was compared in fresh human cadavers. In type 4 bones, the MkIV Brånemark implant maintained a high primary stability in compare with all other types tested (O’Sullivan and Sennerby 2000). However, when measuring the area of these white layers, it was found that self-tapping straight implant of MK3 had the smallest, followed by ST, BL, TE, and self-tapping tapered implant of MK4. Therefore, this result could be the implant macro-design (e.g. tapered and straight) effect to IT than cutting chamber. This order correlates with the implant torque values, suggesting that the size of the white layer is closely related to insertion torque values. It is presumed that the white layer occurs when the cell structure of the artificial bone is damaged by an external force, with variations in reflection factor and refractive index causing the area to become cloudy. Therefore, the location of the white layer is thought to correspond with the site of stress concentration. In addition, if the size of the white layer does indeed correspond with stress, it could be used to indicate the location, direction, and magnitude of force acting on the artificial bone. The present study showed that the IT and white layer increased according to the tapered portion, resulting in a positive correlation. In other words, the primary stability was shown to be highly dependent on the implant design.

Observing the interface between the threads and artificial bone in detail, the upper threads closely engaged the artificial bone, whereas the under did not contact the artificial bone closely, resulting in the presence of a gap. Based on this observation, it is believed that primary stability of the implants was mainly dependent on the friction between the upper surface of the threads and the bone. Potential causes for the gap under to the threads are as follows. During insertion, a rotational force is used to extend the implant into the artificial bone, resulting in slight expansion of the implant as the under thread surfaces rotate and compress the artificial bone upon contact. However, once the rotational force disappears, the implant shrinks by elastic recovery. This shrinkage results in a separation between the under thread surfaces and the bone. In ST and MK3, the parallel implant groups, the threads contacted the artificial bone at the neck. This was a characteristic finding of the parallel implants as it was not observed with tapered implants. This gap was slightly smaller than that noted between the artificial bone and the threads positioned further apically. It is suggested that the elastic recovery force holed the threads and enhanced retention of the implant. The implant platform in MK3 and MK4 engaged the artificial bone closely and no gap was seen. However, white layers such as those around the threads were not observed. The insertion torque-time curves showed that a greater torque values was generated when the platform contacted the bone. This is presumably because the surface where the platform contacted the bone was large and flat, and therefore the force was dispersed and did not create sufficient plastic deformation to destroy the foam. For self-tapping implant of MK3 and MK4, it was confirmed that the bone debris, which occurred during tapping, were distributed over the contacting interface. This study revealed that most of the bone debris was accumulated in the chamber, although some were simultaneously accumulated in the gap between the threads and artificial bone. Therefore, it is thought that a greater decrease in reverse torque occurred because of the bone debris on the contact interface being gradually fragmented and flattened. One of the causes early lost might flattening of the irregularities of the contact area.

## Conclusions

The limitation of this study is that only the correlations of mechanical retention and implant design were evaluated, whereas in clinical situations many biological factors affect primary stability. However, the novel direct observation method of the implant/artificial bone interface is a simple and useful that enables the identification of the area where implant retention occurs. Artificial bone composed a white layer at the site of stress concentration during implant placement; therefore, sites loaded by stress were identified and the magnitude of the stress was quantitatively estimated.

## Abbreviations

PUF: polyurethane foam
ST: standard RN
BL: bone level RC
TE: tapered effect RN
MK4: Brånemark MKIV
MK3: Brånemark MKIII
